# Association of *GAK* rs1564282 With Susceptibility to Parkinson’s Disease in Chinese Populations

**DOI:** 10.3389/fgene.2021.777942

**Published:** 2021-11-18

**Authors:** He Li, Chen Zhang, Yong Ji

**Affiliations:** ^1^ Tianjin Key Laboratory of Cerebrovascular and of Neurodegenerative Diseases, Department of Neurology, Tianjin Huanhu Hospital, Tianjin, China; ^2^ Tianjin Key Laboratory of Cerebrovascular and of Neurodegenerative Diseases, Department of Neurosurgery, Tianjin Huanhu Hospital, Tianjin, China

**Keywords:** Parkinson’s disease, genome-wide association study, GAK, rs1564282, Chinese population

## Abstract

The susceptibility of the *GAK* rs1564282 variant in Parkinson’s disease (PD) in Europeans was identified using a series of published genome-wide association studies. Recently, some studies focused on the association between rs1564282 and PD risk in Chinese populations but with inconsistent results. Thus, we conducted an updated meta-analysis with a total of 7,881 samples (4,055 PD cases and 3,826 controls) from eligible studies. After excluding significant heterogeneity, we showed that the rs1564282 variant was significantly associated with PD in Chinese populations (*p* = 1.00E-04, odds ratio = 1.28 and 95% confidence interval = 1.16–1.42). The sensitivity analysis showed that the association between rs1564282 and PD was not greatly influenced, and there was no significant publication bias among the included studies. Consequently, this meta-analysis indicates that the *GAK* rs1564282 variant is significantly associated with susceptibility to PD in Chinese populations.

## Introduction

Parkinson’s disease (PD) is the second-most common neurodegenerative disease after Alzheimer’s disease (Ascherio and Schwarzschild, 2016). With the widespread use of genome-wide association studies (GWAS), more genetic components of PD have been identified, and potential mechanisms of PD have been uncovered (Nalls et al., 2014; Nalls et al., 2019). In 2009, Pankratz et al. designated *GAK/DGKQ* as a new PD risk region in a Caucasian population (Pankratz et al., 2009). The following GWAS showed that the *GAK* rs1564282 variant was associated with the increasing risk of PD (Spencer et al., 2011). Subsequently, the underlying associations between rs1564282 and PD were investigated in Chinese populations.

Li et al. selected 812 PD patients and 763 control individuals from west China and first corroborated that rs1564282 was associated with PD in a Chinese population (*p* = 0.017) (Li et al., 2012). Then a meta-analysis using a European population reached a similar conclusion (Li et al., 2012).

In 2013, Chen et al. recruited 376 PD patients and 277 healthy controls from west China and identified an association between rs1564282 and PD (Chen et al., 2013). The presence of rs1564282 was reported to significantly increase the risk of PD progression (Chen et al., 2013). However, Lin team and Tseng team evaluated Chinese populations from Taiwan and Singapore respectively and demonstrated no association between rs1564282 and PD (Lin et al., 2013; Tseng et al., 2013).

In 2015, Yu et al. analyzed 534 PD patients and 435 neurologically healthy controls from west China and found that rs156428 was significantly associated with PD (Yu et al., 2015).

The inconsistent association results from the previous studies may be due to at least two reasons: genetic heterogeneity and small sample sizes. Firstly, genetic heterogeneity among the previous studies may lead to the inconsistency. Although all the previous studies included Chinese populations, their population compositions (or structures) and geographical environment at largely varied. In other words, the associations between the risk variants and PD may be different among different populations. Secondly, the smaller sample sizes of the previous studies may also contribute to the inconsistency. In these previous studies, Tseng et al. recruited 978 and 777 samples from Taiwan and Singapore population respectively while the Tian et al. used 2049 individuals for analysis. The results of these studies showed that larger sample sizes provided greater power in discovering significant genetic associations. Because of the inconsistent results, the association between rs1564282 and PD in Chinese populations needs further research. Thus, we conducted a new meta-analysis to investigate the association between rs1564282 and PD via combining previous case–control cohort data.

## Materials and Methods

### Systemic Literature Search

A systemic literature search was performed in four databases: PubMed (http://www.ncbi.nlm.nih.gov/pubmed), Google Scholar (https://scholar.google.com/), China National Knowledge Infrastructure (CNKI, http://www.cnki.net/) and Wanfang Medicine database (http://www.wanfangdata.com.cn/). We screened all the relevant studies using the following terms: “Parkinson’s disease”, “GAK” and “Chinese or China”. Literature published before July 31, 2021 was selected. The detailed content of the inclusion and exclusion criteria is given in the Study Selection section.

### Study Selection

The eligible studies satisfied the inclusion criteria: 1) case–control designed studies in humans, 2) studies calculating the association between rs1564282 variant and PD and 3) studies providing the number of rs1564282 genotypes or adequate data for the calculation of the odds radio (OR) and a 95% confidence interval (CI). Studies that did not satisfy the inclusion criteria were excluded.

### Data Extraction

Two investigators independently extracted the following available data from studies: 1) name of the first author; 2) year of publication; 3) population of study; 4) numbers of PD cases and controls; 5) genotype distribution of rs1564282 in cases and controls; and 6) OR with 95% CI or data for calculating OR and 95% CI.

### Genetic Model

The additive genetic model was used to estimate the association between rs1564282 and PD: the T allele versus the C allele.

### Statistical Analysis

The Hardy–Weinberg equilibrium (HWE) of rs1564282 in the control for each study was calculated respectively with a chi-squared test at *p* < 0.001. We conducted the heterogeneity test using Cochran’s Q test and *I* ^2^ statistic (Liu et al., 2013). The Q statistic follows a χ^2^ distribution with k−1 degrees of freedom (k means the number of researches selected in calculation). The *p*-value of Cochran’s Q test <0.1 means a significant heterogeneity exists among the studies. The statistic *I* ^2^ ( $I^{2}=\frac{Q-\left(k-1\right)}{Q}\times 100\%$ ) reflects the percentage of variation across studies caused by heterogeneity. *I* ^2^ ranges between 0 and 100% (*I* ^2^ = 0–25%, 25–50%, 50–75% and 75–100%), with a higher percentage indicating a greater degree of heterogeneity (Liu et al., 2013; Liu et al., 2017). If there was a large amount of heterogeneity (*p* < 0.1 of Q statistic and *I* ^2^ > 50%), a random-effect model was used for meta-analysis, otherwise a fixed-effect model was chosen. The statistical significance of OR was calculated utilizing a Z-test, with *p* < 0.05 considered significant. We completed the sensitivity analysis through removing any study from the included studies in turn to evaluate the influence of each study on pooled OR and related *p*-value (Liu et al., 2017). Publication bias was estimated by a funnel plot. The regression method propounded by Egger was utilized to test publication bias of the selected studies (Egger et al., 1997). The significant threshold was 0.01. All statistical computations were performed utilizing R (http://www.r-project.org/).

## Results

### Systematic Literature Search

**FIGURE 1:**
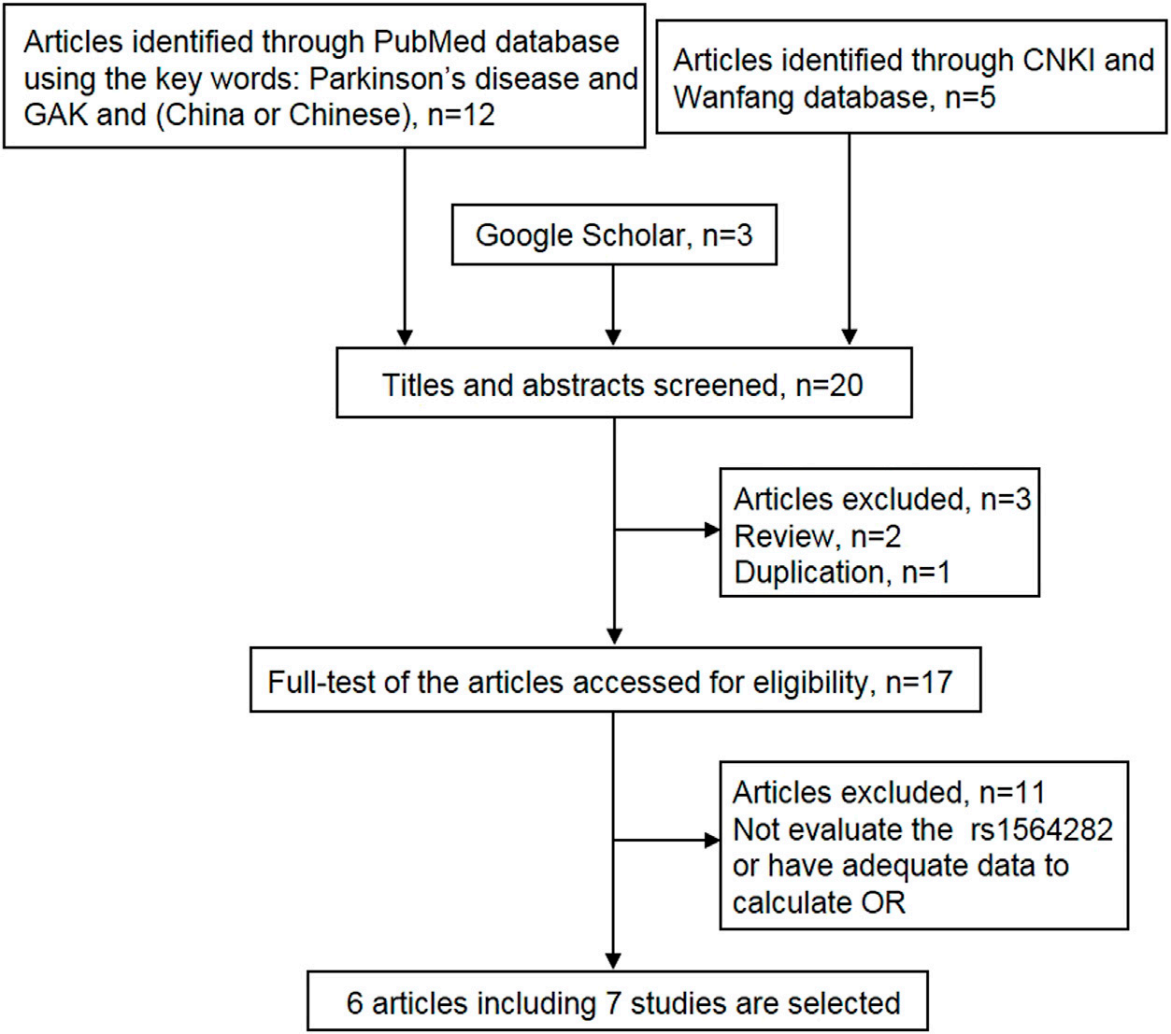
Flow chart of the literature search process.

Utilizing our literature search methods, we obtained 20 articles from four databases (Figure 1). Firstly, three articles were removed due to duplication or being a review. Subsequently, 11 articles were excluded because they did not estimate the association between rs1564282 variant and PD or have sufficient data to compute OR. Finally, six articles that included seven studies, with a total of 4,055 PD patients and 3,826 controls, were selected for the meta-analysis. Detailed characteristics of the eligible studies are listed in Table 1.

**TABLE 1:** Characteristics of seven eligible studies on the association between rs1564282 and PD.

Study	Population	Case	Control	HWE in control	Case genotypes			Control genotypes		
					CC	CT	TT	CC	CT	TT
^a^ Tian. (2012)	South China	1,019	1,030	0.11	814	186	19	866	152	12
^b^ Li et al. (2012)	West China	812	762	0.22	616	183	13	616	142	4
^a^ Chen et al. (2013)	West China	376	277	1	285	81	10	227	48	2
^a^ Lin et al. (2013)	Taiwan	448	452	0.60	341	97	10	363	85	4
^b^ Tseng et al. (2013)	Taiwan	483	495	0.37	381	97	5	387	104	4
^b^ Tseng et al. (2013)	Singapore	388	389	0.095	306	77	5	311	77	1
^a^ Yu et al. (2015)	West China	529	421	0.046	385	132	12	331	89	1

HWE: Hardy–Weinberg equilibrium.

a Study tested multiple SNPs including the GAK rs1564282 with PD in Chinese populations.

b Study only tested the association of one SNP (GAK rs1564282) with PD in Chinese populations.

### HWE and Heterogeneity Test

We evaluated the HWE of rs1564282 in controls for each study respectively. We did not find significant deviation from HWE at *p* < 0.001. Neither Cochran’s Q test nor *I* ^2^ statistic identified significant heterogeneity of rs1564282 polymorphism among the seven studies in Chinese populations (*p* = 0.44 and *I* ^2^ = 0%).

### Meta-Analysis

**FIGURE 2:**
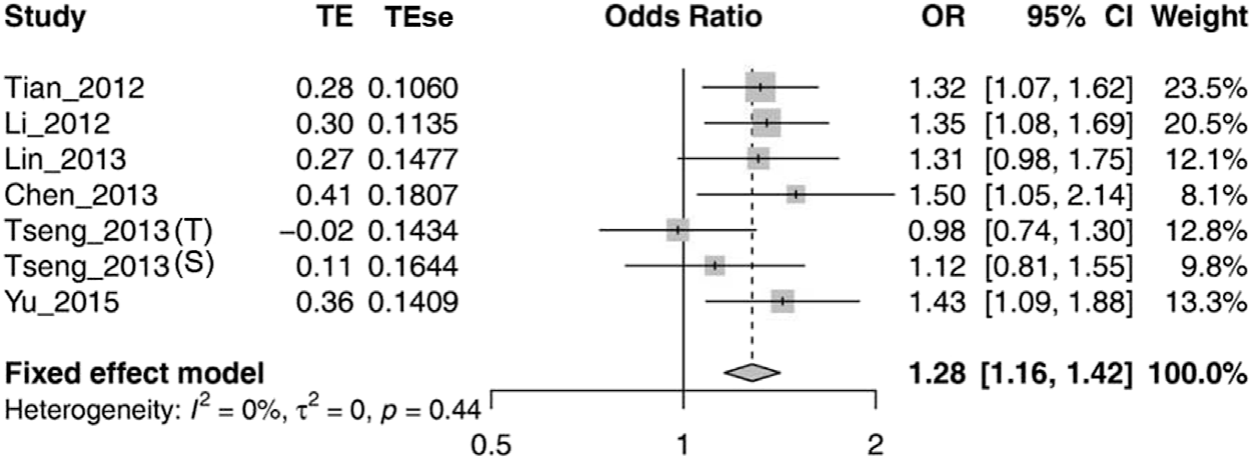
Forest plot for meta-analysis of the association between rs1564282 polymorphism and the risk of PD. Tseng_2013 (T) stands for the Taiwan population in the Tseng_2013 study. Tseng_2013 (S) represents the Singapore population in the Tseng_2013 study. TE = Treatment Effect; TEse = Treatment Effect standard error; CI = Confidence Interval.

Because there was no significant heterogeneity of rs1564282 polymorphism, we computed the general OR with 95% CI using a fixed-effect model. The meta-analysis results demonstrated significant association between *GAK* rs1564282 and PD with *p* = 1.00E-04, OR = 1.28 and 95% CI = 1.16–1.42. More information on the meta-analysis results are shown in Figure 2.

### Sensitivity Analysis and Publication Bias Analysis

**FIGURE 3:**
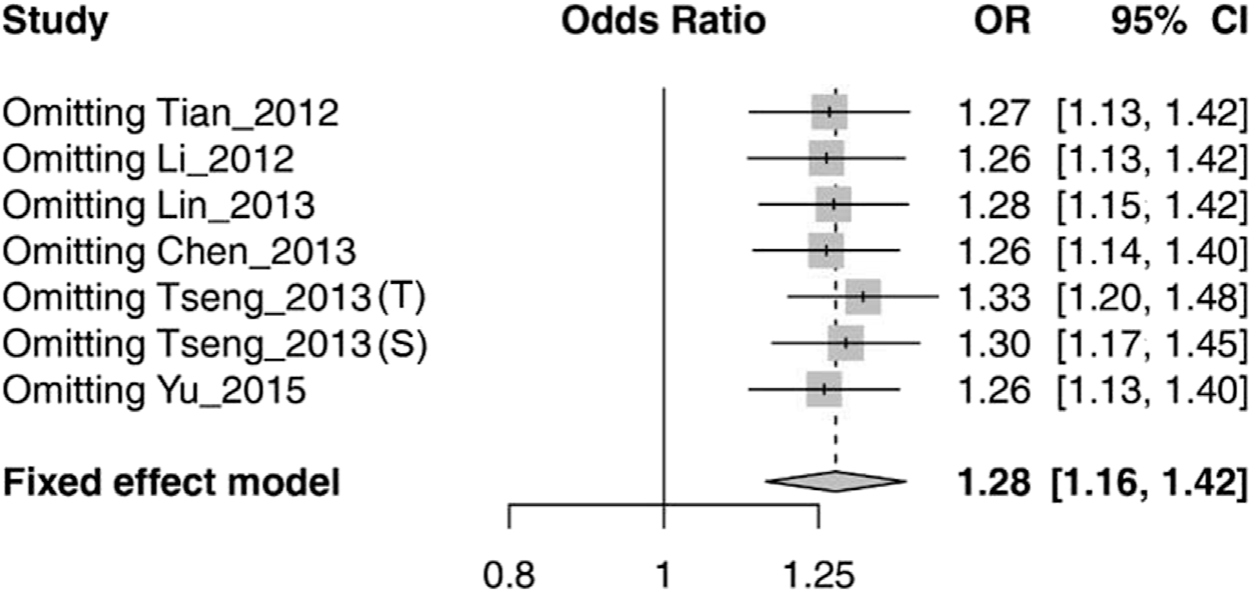
Sensitivity analysis of meta-analysis by omitting every study in turn. Tseng_2013 (T) stands for the Taiwan population in the Tseng_2013 study. Tseng_2013 (S) represents the Singapore population in the Tseng_2013 study. CI = Confidence Interval.

The sensitivity analysis was conducted by removing each study at a time. We found that omitting any eligible study did not substantially influence the overall association between rs1564282 and PD (Figure 3).

**FIGURE 4:**
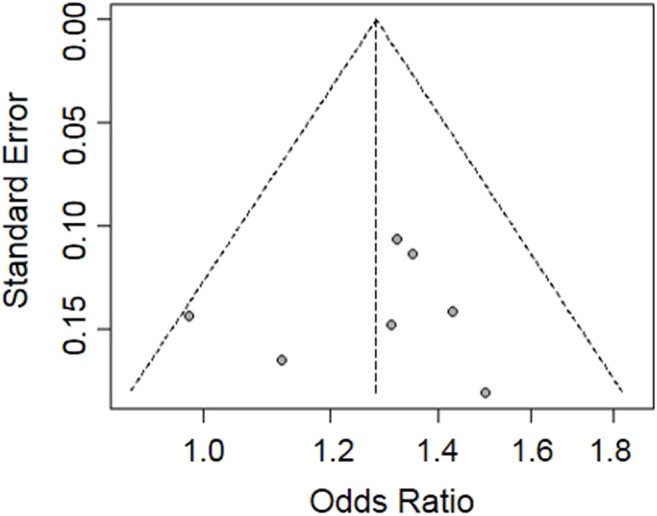
Funnel plot for publication bias analysis of the eligible studies evaluating the relationship between rs1564282 polymorphism and the risk of PD. The *x-*axis and *y-*axis represent the ORs and standard errors for every eligible study, respectively.

The funnel plot was used to estimate the publication bias of the included studies. The shape of the funnel plot was symmetrical and inverted (Figure 4). The regression test showed no significant publication bias among the seven included studies in this meta-analysis (*p* = 0.77).

## Discussion

The genetic association between the *GAK* rs1564282 and PD was first reported in a familial PD GWAS and replicated by following studies in European populations (Pankratz et al., 2009; Hamza et al., 2010; Lill et al., 2012; Nalls et al., 2014). Furthermore, the underlying mechanism of *GAK* in PD has been explored. *GAK* is ubiquitously expressed and participates in various biological processes such as clathrin-mediated membrane traffic and hepatitis C virus entry (Olszewski et al., 2014; Neveu et al., 2015).

In the pathogenesis of PD, rs1564282 was significantly associated with a higher expression level of α-synuclein expression (encoded by *SNCA* gene) in the cortex of PD cases than controls using microarray data (Dumitriu et al., 2011). Dumitriu et al. further investigated the interaction between *GAK* expression and SNCA (Dumitriu et al., 2011). They executed small interfering RNA knockdown of *GAK* in HEK293 cells that overexpressed the SNCA protein and reported that lack of *GAK* expression increased the cytotoxicity based on the overexpression of a-synuclein (Dumitriu et al., 2011).

In addition to the synergistic action with SNCA, evidence also showed that *GAK* impacted the leucine-rich repeat kinase 2 (LRRK2) by forming a complex (Beilina et al., 2014). The gene for LRRK2 has been identified as risk both for monogenic and sporadic PD (Gasser, 2009; Sharma et al., 2012). Beilina et al. utilized the protein–protein arrays to explore the potential interaction mechanisms of LRRK2 in PD pathogenesis (Beilina et al., 2014). The results indicated that *GAK* was a part of a LRRK2-related complex that helped the autophagy–lysosome system to clean vesicles from the Golgi (Beilina et al., 2014).

Nagle’s team conducted deep RNA sequencing in human brain tissue from dead PD patients (Nagle et al., 2016). Compared with controls, *GAK* was a unique gene in the 4p16.3 region which had significantly increased expression in PD after adjustment (q value = 4.80E-09) (Nagle et al., 2016). Song et al. studied the function of *auxilin*, the *Drosophila* GAK homolog, via an *in vivo* model (Song et al., 2017). Through systematic experimentation, auxilin was identified as playing a vital role in PD pathogenesis (Song et al., 2017). Researchers proved that reduced auxilin expression resulted in the progressive loss of dopaminergic neurons (Song et al., 2017). Furthermore, the concurrence of reduced auxilin expression and increased SNCA expression accelerated the early death of dopaminergic neurons (Song et al., 2017). Recent evidence showed that *GAK* was one candidate PD gene that had association with N^6^-methyladenosine modification (Qiu et al., 2020).

So far, PD GWASs and relevant large-scale meta-analyses have identified tens of risk loci in European population (Hamza et al., 2010; Nalls et al., 2011; Nalls et al., 2014; Nalls et al., 2019). As a vital part of the world population, Chinese population accounts for a certain proportion of global PD patients. Strong evidence provided by Foo team identified that *SNCA*, *LRRK2* and *MCCC1* genes had genome-wide significant associations with PD susceptibility in both Chinese and European population (Foo et al., 2017). In the analysis of risk loci, they inferred that *MAPT* and *GBA* genes might be “European-specific variant loci” (Foo et al., 2017). In subsequent studies, some PD risk loci with genome-wide significance identified in European population had been confirmed to have association in Chinese population, for example *GALC*, *IL1R2*, *SATB1*, *BIN3* and *COQ7* genes (Li et al., 2018; Chen et al., 2019; Hu et al., 2020).

Several studies estimated the underlying association between rs1564282 and PD risk in Chinese populations in China and Singapore (Tseng et al., 2013; Yu et al., 2015). However, the results of these studies were not consistent. We integrated the pooled data of previous studies and conducted a new meta-analysis with 4,055 PD patients and 3,826 controls in all. Firstly, we identified that there was no significant genetic heterogeneity of rs1564282 in the included Chinese populations. Subsequently, the meta-analysis using a fixed-effect model showed a significant association between rs1564282 and PD in Chinese populations. Finally, we performed sensitivity and publication bias analysis. Results showed that the association between rs1564282 and PD was not greatly influenced substantially and that there was no significant publication bias among the eligible studies. In conclusion, our meta-analysis provides good evidence on the risk of the *GAK* rs1564282 variant on PD in Chinese populations.

## Data Availability

The original contributions presented in the study are included in the article/Supplementary Material, further inquiries can be directed to the corresponding author.

## Abbreviations

CI, confidence interval; GWAS, genome-wide association studies; HWE, Hardy–Weinberg equilibrium; LRRK2, leucine-rich repeat kinase 2; OR, odds radio; PD, Parkinson’s disease.
